# Unveiling disguised toxicity: A novel pre-processing module for enhanced content moderation

**DOI:** 10.1016/j.mex.2024.102668

**Published:** 2024-03-26

**Authors:** Johnny Chan, Yuming Li

**Affiliations:** University of Auckland, Private Bag 92019, Auckland 1142, New Zealand

**Keywords:** Natural language processing, Text pre-processing, Toxic text detection, Specialis Revelio

## Abstract

This study introduces “Specialis Revelio,” a sophisticated text pre-processing module aimed at enhancing the detection of disguised toxic content in online communications. Through a blend of conventional and novel pre-processing methods, this module significantly improves the accuracy of existing toxic text detection tools, addressing the challenge of content that is deliberately altered to evade standard detection methods.

• Integration with Existing Systems: “Specialis Revelio” is designed to augment popular toxic text classifiers, enhancing their ability to detect and filter toxic content more effectively.
• Innovative Pre-processing Methods: The module combines traditional pre-processing steps like lowercasing and stemming with advanced strategies, including the handling of adversarial examples and typo correction, to reveal concealed toxicity.
• Validation through Comparative Study: Its effectiveness was validated via a comparative analysis against widely used APIs, demonstrating a marked improvement in the detection of various toxic text indicators.

## Graphical abstract

**Figure:**
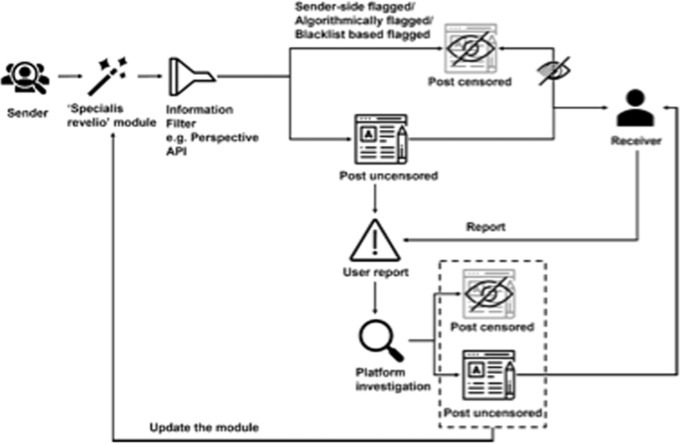

Specifications Table

**Table:** 

Subject area:	Computer Science
More specific subject area:	Natural Language Processing
Name of your method:	Specialis Revelio
Name and reference of original method:	N/A
Resource availability:	N/A

## 1 Introduction

The advent of the digital age has ushered in an era of unprecedented connectivity, fostering a global exchange of ideas and information. As individuals increasingly immerse themselves in digital ecosystems for educational, professional, and recreational purposes, the influence of online discourse on societal norms and individual behaviours has become a subject of paramount importance. These digital platforms, while serving as catalysts for societal discourse, can also metamorphose into breeding grounds for toxic content, if left unchecked. The propagation of such content can have deleterious psychological and emotional effects on users, particularly those belonging to vulnerable demographics. Moreover, the spread of misinformation and disinformation can incite confusion, fear, and even violence, thereby undermining the societal fabric.

The responsibility of mitigating the dissemination of toxic content often rests on the shoulders of social media companies. Their role extends beyond user protection, encompassing the prevention of their platforms being exploited for the propagation of hate speech, violence, or other harmful activities. However, the current toxic content detection mechanisms employed by these platforms can be fraught with deficiencies, allowing a significant volume of toxic content to evade detection. This is particularly true when users resort to evasion tactics such as the addition of noise or the alteration of characters in toxic words.

The need for robust systems to maintain safe and conducive digital environments is more pressing than ever. This paper aims to critically examine the existing approach to text filtering, delineate an ideal approach, and propose a pathway towards achieving it, leveraging existing processes and tools. A key focus will be on the strategies employed by individuals who intentionally disseminate harmful information to circumvent platform censorship. These tactics often involve the use of additional characters in toxic words, numerical abbreviations, slangs, leetspeak, and other methods designed to evade detection while preserving the toxic intent of the message. The challenge lies in the fact that these tactics, while effective at evading system-prescribed blocking rules, still allow the toxic message to be understood by the reader, thereby causing harm. This paper aims to address this challenge, proposing a novel solution to enhance the efficacy of content moderation and contribute to the creation of safer digital environments.

### 1.1 Understanding the current approach

**Fig. 1:**
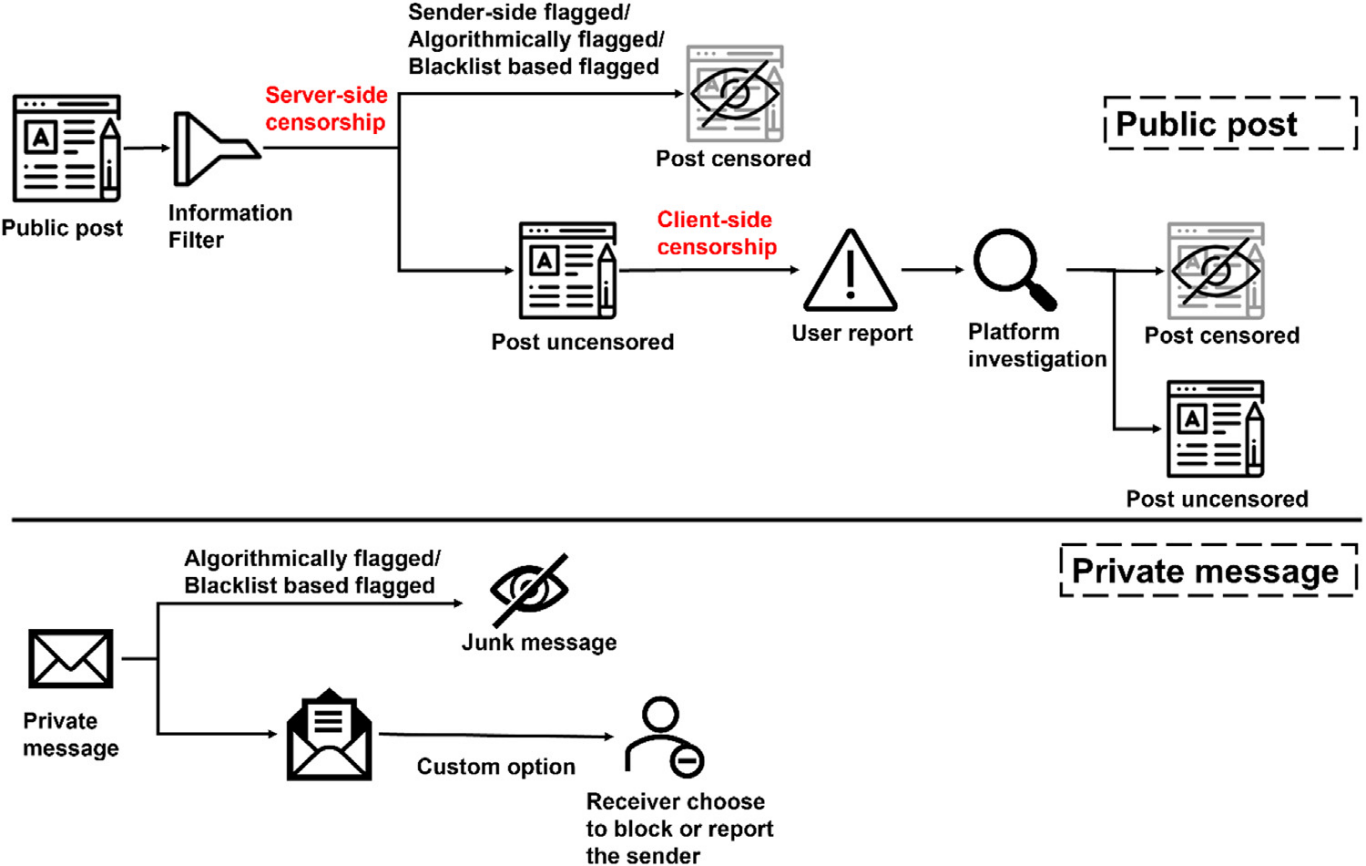
The current approach for toxic content filtering.

The prevalent method for moderating toxic content in digital environments, encompassing both public posts and private messages, is depicted in Fig. 1.

To understand the nuances of this current approach, we refer to the seminal work by Ruan et al. [1], which employed reverse engineering to elucidate the mechanisms of social media censorship, using the case study of COVID-19. Their findings suggest that the existing mechanisms can be broadly categorized into two types: server-side censorship and client-side censorship. Server-side censorship primarily relies on information filters embedded within the server, which include built-in algorithms or blacklists. These tools facilitate preliminary screening and subsequent blocking of toxic content. However, posts that evade this initial layer of censorship can still be manually reported by users upon receipt. Following an investigation by the platform, the post is then classified as toxic or non-toxic, and appropriate action is taken. The framework for detecting harmful content in private messages is somewhat similar to that of public posts, albeit with a few key differences. Given that private messages are typically exchanged between a sender and a receiver, the receiver has the autonomy to judge the content from their perspective, without the need for third-party intervention by the platform.

While the approach outlined in Fig. 1 encapsulates the majority of censorship mechanisms currently in use, it is not impervious to evasion. There exist loopholes that can be exploited by users with malicious intent, thereby undermining the efficacy of these mechanisms. The prevalent approach to moderating toxic content, relying on server-side and client-side censorship mechanisms, presents notable disadvantages. Despite preliminary screening through algorithms and user reports, it fails to comprehensively catch or mitigate all toxic content, leaving exploitable loopholes for malicious users.

### 1.2 Tricks on bypassing censorship

Regrettably, a subset of users, driven by malicious intent, often resort to sophisticated tactics to circumvent algorithmic censorship implemented by digital platforms. These tactics can include the addition of noise or the deliberate alteration of characters within toxic words. Despite these modifications, the harmful intent of the message remains perceptible to the recipient, leading to potential harm. When such malicious activities reach a certain scale, they can even inspire the development of algorithms or APIs specifically designed to bypass censorship mechanisms, as highlighted by Hiruncharoenvate et al. [2]. While these algorithms may not inherently possess malicious attributes, their misuse by malevolent users can inflict irreversible harm on recipients and subvert the original purpose of social media platforms.

This paper draws inspiration from the pioneering work of Gil et al. [3], who trained a model to simulate a white-box attack aimed at exploiting the vulnerabilities of Google's Perspective API. The Perspective API is a very popular tool that uses machine learning to identify and filter toxic comments in online discussions [4,5]. The authors generated adversarial samples through an optimization process involving gradient descent, which were then used to attack Perspective's toxicity classifier. This approach resulted in a misclassification rate of 42% for the generated adversarial samples by the API. For instance, the authors used the adversarial sample “to be driven away and dke” in place of the original sample “to be driven away and die” to test the Perspective API. The final toxicity score was reduced from 0.82 to 0.32, effectively evading platform censorship. The generation of these adversarial samples was primarily based on the HOTFLIP method [6], which modifies input samples according to the gradient through character-level changes such as swapping, inserting, or deleting. At its core, HOTFLIP employs a gradient-based approach to determine the impact of various modifications to the text, such as flipping a character (changing one character to another), adding new characters, or removing existing ones. By analysing the model's gradients with respect to the input text, HOTFLIP identifies which changes would have the maximum impact on the model's output with the least amount of alteration to the original text.

Suppose the input original sentence is$X=[\left(x_{11},x_{1n}\right);..\left(x_{m1},..x_{mn}\right)]$ where *n* is the length of the longest word among all words, and *m* is the number of words in sentence *X* encoded as 1-hot vectors. HOTFLIP calculates the best possible character to be flipped through forward pass and backward pass. For example, if the *i^th^* character needs to be changed from *o* to *c*, then the transformation can be represented by the following vector:

$$\vec{v_{c}^{i}}=\left(\ldots ,{\left(0,\ldots ,-1,\ldots ,1\ldots ,0\right)}_{i},\ldots \right)$$

Where the positions of −1 and 1 represent the original positions of the characters *o* and *c* respectively. Then the loss of this flip is calculated by $\nabla_{X}L\left(X,Y\right)$ with directional derivative along $\vec{v_{c}^{i}}$:

$$\nabla_{\vec{v_{c}^{i}}}L\left(X,Y\right)=\nabla_{X}L\left(X,Y\right)\cdot \vec{v_{c}^{i}}$$

in which *L*(*X,Y*) is the loss function for input *X* respect to label *Y*. Then the gradient is used with respect to the input *X* to maximize the estimates of flipped *o* and *c*:

$$\left[\nabla L\left(X,Y\right)\cdot \vec{v_{c}^{l}}\right]=\left[\frac{\partial L}{\partial X_{i}^{c}}-\frac{\partial L}{\partial X_{i}^{o}}\right]$$

When the maximum value is taken, it is the best flip of the word, and the adversarial example $\underset{‾}{X}$ is finally obtained.

Adversarial examples have recently gained considerable attention in the field of Natural Language Processing (NLP). Despite the robust generalization capabilities of deep neural network models, they exhibit a high degree of vulnerability to adversarial attacks. This susceptibility was highlighted by Szegedy et al. [7], who found that the introduction of subtle noise to samples used as input for deep models could lead to misclassifications. Specifically, these 'fooling examples' were assigned high confidence scores by the model, resulting in erroneous outputs. This vulnerability is a prevalent issue in toxic content filters that are based on deep neural network algorithms.

### 1.3 Specialis Revelio

In light of the limitations of existing tools in content moderation, there is a pressing need for more robust and resilient solutions. While Perspective API and similar tools have made significant strides in detecting toxic content, they are primarily effective on short text spans and lack the ability to model context throughout a longer conversation. This means that more subtle forms of harassment can slip through the cracks. Furthermore, these solutions are not robust against circumvention attempts, where a malicious user may manipulate the text by introducing spelling errors, irregular punctuations, or other alterations while preserving the original toxic intent. These manipulations can significantly reduce the confidence of the models in recognizing toxicity.

**Fig. 2:**
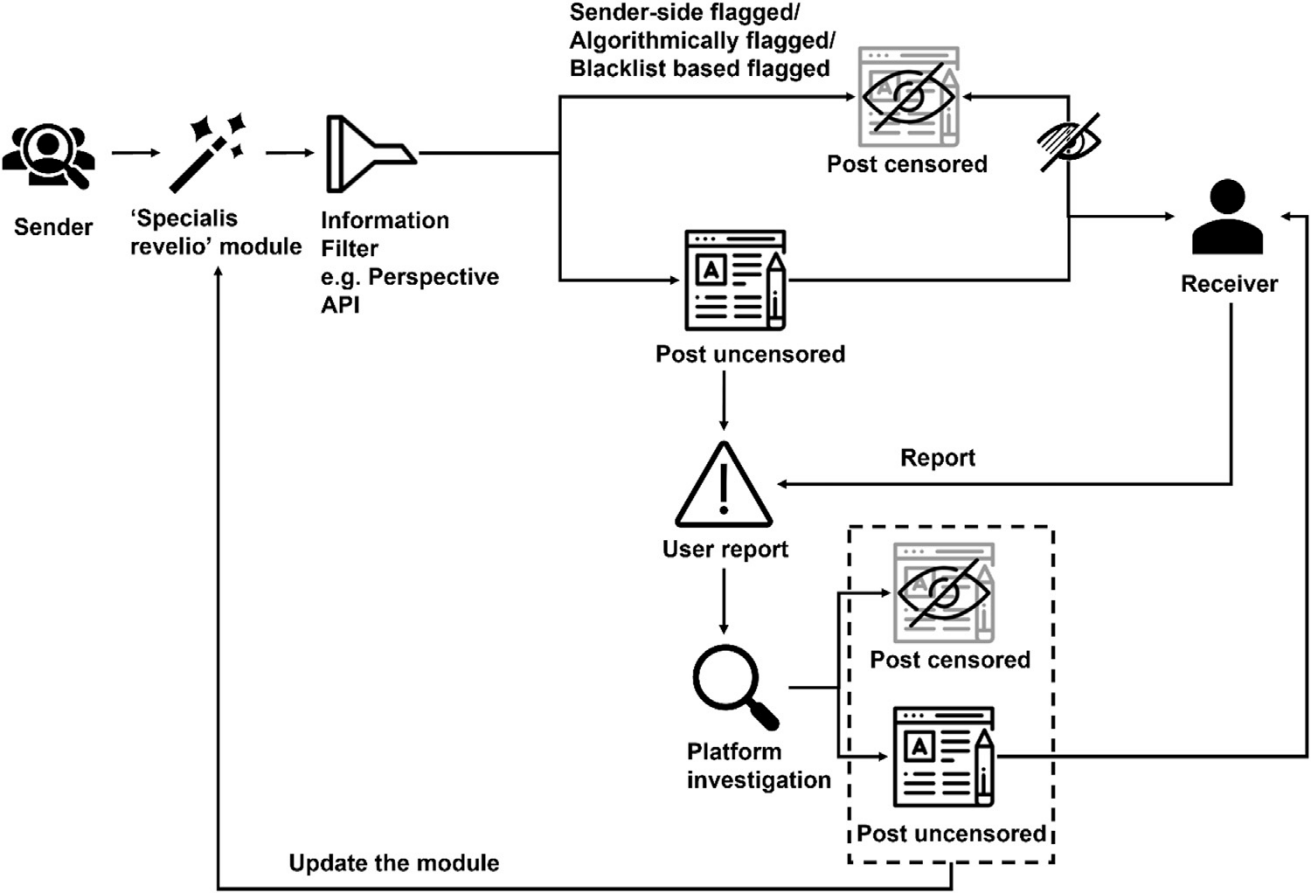
An ideal approach for toxic content filtering.

Addressing these challenges, we propose a novel approach to enhance the efficacy of toxic text detection services. While addressing the lack of contextual awareness is technically challenging at present, the circumvention problem can be mitigated. We propose a simple toxic text filtering pipeline, as shown in Fig. 2. This approach leverages existing toxic text classifiers and augments them with a pre-processing module to counteract circumvention attempts.

In this context, we introduce Specialis Revelio, a pre-processing module named after a charm in the Harry Potter series used to reveal hidden spells or jinxes. Much like its namesake, our Specialis Revelio aims to uncover hidden toxic content. This module incorporates traditional pre-processing techniques and employs the concept of adversarial examples in a novel way. It aims to restore artificially altered toxic text through typo correction, thereby revealing toxic content that was designed to evade censorship. By enhancing the filtering accuracy of toxic content detection APIs, Specialis Revelio represents a promising step towards safer digital environments.

## 2 Related work

The burgeoning issue of toxic content and harmful speech on digital platforms has garnered significant scholarly attention in recent years. The primary research methodology in this domain involves the development of text classifiers based on deep learning models to categorize and identify toxic content. This approach has been explored extensively in the literature, with studies employing a variety of deep learning models such as convolutional neural networks [8], recurrent neural networks [9], and hybrid models [10] to classify and recognize text. Zhao et al. [11] further advanced this field by introducing pre-trained language models to improve the accuracy of toxic comment classification. Their comparative study of three popular language models, namely BERT [12], RoBERTa [13], and XLM RoBERTa [14], concluded that BERT and RoBERTa generally outperform XLM RoBERTa in the classification of toxic content. This research underscores the potential of pre-trained language models in enhancing the efficacy of toxic content detection.

However, deep learning-based models are often criticized for their lack of interpretability and their 'black-box' effects. Addressing this concern, Mahajan et al. [15] proposed an interpretable harmful comment classification model based on pre-trained language models and gated recurrent units (GRU). They provided an intuitive explanation for their model using the Local Interpretable Model-agnostic Explanation (LIME) method [16], contributing to the ongoing discourse on the interpretability of deep learning models. One of the challenges in the detection of harmful speech and toxic content is the relative scarcity of training data, as compared to general classification data. This is due to the extreme language expressions often found in harmful speech and toxic content. To address this issue, Rastogi et al. [17] proposed a data augmentation approach, enhancing the data based on Easy Data Augmentation (EDA) and Backtranslation. Their findings suggest that data augmentation can significantly improve the performance of classifiers in toxic text classification tasks.

Furthermore, the heterogeneity of harmful text datasets, stemming from different languages or sources, presents another challenge. These datasets often have different classification indicators, complicating the task of toxic content detection. Risch et al. [18] proposed a data integration tool that amalgamates datasets from over 30 different sources, significantly alleviating the problem of data scarcity. This tool also provides an overview of the properties of different datasets, enabling users to select training and testing data more flexibly. This represents a significant advancement in the field, offering a promising solution to the challenges posed by data scarcity and heterogeneity.

## 3 Conceptualising an ideal approach

In the contemporary digital landscape, numerous platforms have implemented NLP systems capable of understanding conversational context and automatically detecting toxic content, harassment, hate speech, and misinformation. Despite the rapid advancements in NLP systems across various tasks [19], we have yet to reach a stage where these context-aware systems can be widely deployed. This is not due to a technological deficit, as evidenced by Google's chatbot Meena [20], which can track conversational context. Instead, the primary barriers are the computational resources and extensive training data required.

An optimal approach would be capable of tracking the evolution of conversations and detecting toxicity in real-time. Several text-based toxicity detection methods and tools are currently available, including Google's Perspective API, which leverages NLP to identify text-based toxicity in real-time. The benefits of using such a toxicity-detection-as-a-service are clear: it eliminates the computational and data costs associated with developing a similar system from scratch. However, since it is a third-party pre-trained model, there is no potential for future improvement through continued usage.

In Fig. 2, we present an ideal approach for toxic content filtering, which integrates public posts and private messages from the micro perspective of message senders and receivers. As per our earlier discussion, in an improved approach, the information generated by the sender should undergo a comprehensive pre-processing module before being sent to a real-time toxic text detection API like Perspective. This pre-processing module, which we refer to as the Specialis Revelio module, is designed to prevent malicious attempts to bypass censorship.

Once the information has been processed, it is filtered into censored and uncensored posts by the real-time toxic text detection API or tool. Censored posts are blocked or flagged based on the platform's policies, while uncensored posts are directly transmitted to the receiver. As a receiver, one can choose to view or ignore posts that have been automatically flagged as toxic by the filter (the specific functions are determined by platform rules, such as regarding the protection for underage users, the censored posts may be directly blocked). Receivers can also manually report posts that have not been pre-screened, which are then reclassified as censored or uncensored posts following an investigation by the platform. Posts that are not automatically flagged will be used as new samples in the training data of the pre-processing module and the filter module, thereby optimizing and iterating the overall toxic text detection framework.

This approach, compared to traditional methods, adds a double-check module on the sender side and minimizes the potential harm to the receiver caused by malicious text modified using tricks to avoid censorship. In the following sections, we delve into the key components of the pre-processing module in greater detail, discussing the circumvention issues they target and the opportunities and challenges in mitigating them.

### 3.1 Designing an enhanced text pre-processing approach

Text pre-processing, a pivotal step in NLP, involves the cleaning and preparation of text data for subsequent analysis or modelling. The quality of pre-processing directly influences the performance of the subsequent models, adhering to the principle of “garbage in, garbage out”. In the context of online platforms such as social media, text data often contains noise, uninformative elements, and deliberate distractions. When undertaking certain online text processing applications, taking toxic text detection as an example, the extent of text preprocessing often directly determines the quality of the application. Detecting toxic content online faces significant challenges, as traditional methods struggle against users who cleverly disguise their messages to evade censorship. These disguised communications bypass existing filters, reaching recipients with their harmful intent intact. This paper aims to propose an enhanced text pre-processing approach to tackle the sophisticated evasion tactics employed in disseminating toxic content online. The enhanced text pre-processing approach we proposed incorporates both traditional and novel pre-processing steps. We uniquely integrate often-overlooked pre-processing steps such as word-boundary changes and Leetspeak conversion, within a comprehensive pipeline, guided by a set of newly developed rules for the strategic application and combination of both conventional and advanced techniques. Overall, the proposed pre-processing module integrates seven steps: lowercasing, stemming, stopword and special character removal, word-boundary changing, slang and Leetspeak removal, and GPT-3 based misspelling correction, to robustly identify and mitigate disguised toxic content online.

### 3.2 Traditional pre-processing steps

#### Lowercasing

This step standardises text data to ensure uniformity, addressing the case sensitivity of many word embedding methods and thereby reducing the complexity of subsequent analyses. In the realm of NLP, text data is typically converted into numerical vectors that can be processed by machine learning algorithms. However, word embedding methods such as Word2Vec [21] are case-sensitive, treating “toxic”, “Toxic”, and “TOXIC” as distinct words. This can interfere with subsequent models, reducing the accuracy of the final classification result. Therefore, converting all text to lowercase is a crucial pre-processing step. In the initial phase of our module, we standardise the case by transforming all text to lowercase. This normalisation is executed via the text.lower() method, a built-in function in Python's standard library, to eliminate discrepancies caused by case sensitivity, which is crucial for uniform text analysis.

#### Stemming

By reducing words to their base or root form, stemming helps in diminishing the redundancy of the input data, which in turn streamlines the processing and analysis of text data by decreasing its variability. Stemming aims to reduce the redundancy of word vectors by converting different forms of a word into its base form. For instance, “ate”, “eating”, and “eaten” would all be converted to “eat”. This not only retains the original meaning but also reduces noise during embedding, thereby enhancing the efficiency of the model. We employ the Snowball Stemmer algorithm, an extension of the Porter Stemmer [22], facilitated by the Natural Language Toolkit (NLTK). This algorithm adeptly reduces words to their base or root form, streamlining the dataset by minimising lexical diversity without losing significant semantic meaning.

#### Stopword removal

Removing stopwords, which are commonly occurring but minimally informative words, is essential for focusing the analysis on the more meaningful content of the text, thereby enhancing the efficiency of the following models. Stopwords, such as “a”, “the”, and “of”, often lack significant meaning and appear more frequently than other words in the text. To prevent these words from increasing the dimensionality of text features, they are typically removed during pre-processing. Utilising the comprehensive stopword lists provided within the NLTK library, our module systematically eliminates stopwords from the text. These words, though frequent, offer little to no value in understanding the context of the content, thus their removal significantly declutters the textual data for analysis.

#### Special character removal

Special character removal is crucial for maintaining the clarity and relevance of the data, ensuring that the models focus on the textual content that carries semantic weight. Text data obtained from social media often contains special characters, emojis, and HTML tags. These elements can affect the efficiency of subsequent model classification and can be used by malicious users to bypass censorship mechanisms. Therefore, our pre-processing module includes the removal of special symbols, accented characters, HTML, and other non-letter characters, identified through their ASCII codes ranging from 33 to 47, 58 to 64, 91 to 96, and 123 to 126. We address the purification of text by removing non-contributory special characters—via sophisticated regular expression (regex) patterns in NLTK. This step is pivotal in cleansing the text of artefacts that could potentially skew analysis outcomes.

### 3.3 Novel pre-processing steps

#### Word-Boundary changing

In the context of online communication, users often manipulate the boundaries of words to bypass automated content moderation systems. This manipulation, referred to as word-boundary changes by Gröndahl et al. [23], involves the addition or removal of whitespace within sentences. For instance, the sentence “They are liberal idiots who are uneducated” is identified as 96% likely to be toxic by Perspective. However, when the spaces are removed and each word is capitalized with the first letter, forming “They are liberal idiots who are uneducated”, the likelihood drops to 21%. Adding extra spaces between each letter, as in “They are liberal idiots who are uneducated”, results in a toxicity likelihood of 28% [24]. While these manipulations make sentences more difficult for humans to read, the original meaning remains understandable. This is particularly problematic on platforms like Twitter, where hashtags, which cannot contain spaces, are often used to disseminate harmful content.

To counteract this, one approach is to use word segmentation, which employs a probabilistic language model built from a large corpus to determine the most probable way to split a sentence with no whitespace using dynamic programming [25]. If a sentence is detected to have an anomalous number of whitespaces, all whitespaces can be stripped from the sentence and it can be run through the segmentation algorithm. This approach can perfectly reconstruct the original sentence in cases where the words are correctly spelled and are part of the corpus used to build the probabilistic model. However, it may fail if this is not the case, making it crucial to address any spelling errors, abbreviations, or other transformations first.

To directly address the manipulation challenge, our module employs the SymSpell library [26], our module corrects errors in word boundary formation, including but not limited to, misconcatenations and inadvertent whitespace omissions. SymSpell's advanced algorithm enhances text readability and analysis accuracy by ensuring proper word segmentation based on probabilistic models of language use.

#### Slang & Leetspeak removal

It is possible to use various linguistic transformations to evade automated content moderation systems. One such transformation involves the use of numerically abbreviated words, such as “2night”. To address this, one could employ phonetic encodings, such as Soundex, the International Phonetic Alphabet, Metaphone, or Double Metaphone [27]. This process first converts the number to its English spelling, for instance, “twonight,” before encoding the resultant string phonetically. A lookup table of phonetic encodings to English words can then be used to convert the phonetic encoding directly into a correctly spelled English word. While this approach works for simple cases, it can result in mistranslations where the phonetic encoding is replaced with an incorrect word. This is undesirable as it is crucial for the pre-processing model to preserve the original meaning of the text when normalising the input. To mitigate this, a probabilistic model, such as a Hidden Markov Model, can be introduced to model the transition between words in a sentence, thereby guiding the most probable word choice in a given sentence.

Leetspeak, another linguistic transformation, substitutes letters for similar-looking symbols or strings of symbols. This presents a more challenging problem as it is difficult to determine what letter a symbol may substitute for. For instance, in the leetspeak transformation of the word “hello” to to “he11o”, it is not immediately clear that the symbol “1” should be swapped out for an “L” as it also visually resembles an upper-case “i”. To address this, a character-level probabilistic model could be utilised, which could recognise that since the two “1”s are preceded by “he” and followed by “o”, they are more likely to be the consonant “l” rather than the vowel “i”. Alternatively, all reasonable substitutions could be performed and the resultant word which is in a corpus of known words or best fits the sentence as determined by a sentence-level probabilistic model could be selected.

A more sophisticated approach would be to utilise machine translation to convert leetspeak to standard English. Prior work has shown success in converting English SMS text into normalised English [28]. However, state-of-the-art machine translation requires large datasets consisting of parallel texts from the original language and the target language. Given that leetspeak is rare on online platforms, such a dataset cannot be naturally sourced. To circumvent this issue, one could simulate leetspeak with random symbol substitutions, thereby transforming a large corpus of natural English text into leetspeak. This parallel dataset could then be used to train machine translation models, resulting in a system robust at normalising leetspeak style text into natural English.

Building on this foundation, our module incorporates an enhanced detection system for slang and leetspeak by integrating a bespoke message slang translator. This component, through a comprehensive mapping and translation mechanism, reverts such expressions to their standard English equivalents, thereby maintaining the analytical integrity of the text.

#### Misspelling correction

In the proposed pre-processing module, we employ a GPT-3 [19] based approach for the correction of misspellings. GPT-3, developed by OpenAI, is a state-of-the-art language model that has demonstrated proficiency in a wide array of natural language processing tasks, including the correction of misspelled words. The methodology of GPT-3 involves the use of advanced language modelling techniques, such as rule-based methods and statistical models, as well as machine learning algorithms. GPT-3 has been trained on an extensive dataset, “Common Crawl”, and other texts from OpenAI, such as Wikipedia entries, which allows it to learn patterns and relationships between words that can be leveraged to improve the correction of misspellings. This is particularly useful when dealing with malicious users who manually introduce noise or use adversarial examples to bypass censorship. The words presented by flipping, adding, and deleting characters often exhibit obvious misspellings or logical errors in the context. Therefore, in this paper, we choose to use GPT-3, which is based on context modelling, as one of the solutions. In the pre-processing module proposed in this paper, we directly call the API of GPT-3 to restore the trick words through text misspelling correction.

Emphasising the utilisation of GPT-3′s vast training and its advanced capabilities allows us to specifically target and correct a wide range of misspellings, including those manipulated with the intent to evade detection. Our module not only addresses common misspellings but also adapts to the nuanced and continually evolving tactics used by individuals to obscure toxic content, ensuring a more secure and reliable online environment.

## 4 Experimental analysis and results

The primary objective of our experimental analysis was to investigate the significance of data pre-processing in the context of toxic content detection APIs. We conducted a comparative study between two widely utilized APIs, namely Detoxify and Perspective API, to identify six toxic text indicators (toxicity, severe-toxicity, obscene/profanity, threat, insult, identity-attack). We also examined the impact of various pre-processing steps on the results of text toxicity attributes. Detoxify, an open-source Python library, is designed to detect toxic language in text. We selected Detoxify for comparison due to its widespread use in harmful text detection.

### 4.1 Comparative experiment settings

The proposed pre-processing module encompasses seven steps, each leveraging the capabilities of various established libraries and methodologies. Lowercasing is straightforwardly performed by applying the *text.lower()* function from Python's standard library to the input text. Stemming, the reduction of words to their root forms via the NLTK library. The removal of stopwords is executed employing the NLTK library's incorporated stopword lists. For the elimination of special characters, which include punctuation marks, symbols, and non-alphanumeric characters, we apply algorithms based on regular expressions. To address issues related to word-boundary errors, such as incorrect word concatenations or omitted spaces, we employ the SymSpell library, which is designed to autonomously detect and rectify such anomalies. To counter the challenge posed by slang and leetspeak within the input text, a message slang translator script is utilized. This script has the ability to identify and translate these vernacular expressions back to their standard English counterparts. For misspelling, we leverage a GPT-3 based approach.

### 4.2 Example sentences and noise introduction

We selected two example sentences from Gil et al.’s article [3] and introduced noise based on real-world scenarios to the original sentences. Through experimentation, we found that in these two examples, where noise was maliciously added to evade censorship, traditional pre-processing processes, such as Lowercasing and Special Character Removal, still failed to identify the toxic information. However, after applying our proposed Novel Pre-processing Steps, there was a qualitative leap in the detection of toxicity. As per the results presented in Table 1, Detoxify API demonstrated higher confidence in identifying toxic attributes of text. For instance, in cases where Examples 1 and 2 were both marked as toxicity in their source paper, after the pre-processing step of 'misspelling correction', Detoxify could elevate the probability of the final toxicity attribute to exceed 0.95. However, Perspective API only achieved an average toxicity attribute probability of 0.8. Even after setting the threshold, the recognition results were the same, but Detoxify provided a more confident and higher probability for the final filter result. However, in terms of sensitivity to toxic text attributes, Perspective API performed better.

**Table 1:** Experimental analysis results.

Detoxify API							
Pre-processing step	Content	Toxic attribute					
		toxicity	severe toxicity	obscene	threat	insult	Identity attack
Example 1							
Raw Text	Decline=IdntVuc!kingThinkSo	0.047	0.001	0.002	0.001	0.001	0.001
Lowercasing	decline=idntvuc!kingthinkso	0.047	0.001	0.002	0.001	0.001	0.001
Special Character Removal	Declineidntvuckingthinkso	0.188	0.001	0.017	0.001	0.004	0.001
Word-Boundary Changing	decline i dnt vucking think so	0.696	0.007	0.620	0.002	0.116	0.004
Slang & Leetspeak Removal	decline i dont vucking think so	0.744	0.006	0.619	0.001	0.098	0.002
Misspelling Correction	decline i dont fucking think so	0.982	0.096	0.962	0.004	0.128	0.003
Stopword Removal	decline dont fucking think	0.989	0.126	0.975	0.004	0.218	0.003
Stemming	declin dont fuck think	0.990	0.126	0.976	0.003	0.288	0.003
Example 2							
Raw Text	<html> \<*p*>IThinkThe1MillionSalesisTotal BullshktThough</*p*></html>	0.011	0.001	0.001	0.001	0.002	0.001
Lowercasing	<html> \<*p*>ithinkthe1millionsalesistotalbull shktthough</*p*></html>	0.011	0.001	0.001	0.001	0.002	0.001
Special Character Removal	ithinkthe1millionsalesistotalbullshktthough	0.021	0.001	0.001	0.001	0.001	0.001
Word-Boundary Changing	i think the 1 million sales is total bullshkt though	0.458	0.003	0.315	0.001	0.055	0.003
Slang & Leetspeak Removal	i think the 1 million sales is total bullshkt though	0.458	0.003	0.315	0.001	0.055	0.003
Misspelling Correction	i think the 1 million sales is total bullshit though	0.960	0.031	0.910	0.001	0.121	0.002
Stopword Removal	think 1 million sales total bullshit though	0.979	0.423	0.928	0.002	0.213	0.002
Stemming	think 1 million sale total bullshit though	0.979	0.039	0.922	0.001	0.259	0.002

Perspective API							
Pre-processing step	Content	Toxic attribute					
		toxicity	severe toxicity	profanity	threat	insult	Identity attack
Example 1							
Raw Text	Decline=IdntVuc!kingThinkSo	0.210	0.023	0.377	0.008	0.064	0.007
Lowercasing	decline=idntvuc!kingthinkso	0.153	0.013	0.187	0.010	0.046	0.013
Special Character Removal	Declineidntvuckingthinkso	0.238	0.025	0.519	0.006	0.045	0.004
Word-Boundary Changing	decline i dnt vucking think so	0.361	0.023	0.101	0.009	0.101	0.012
Slang & Leetspeak Removal	decline i dont vucking think so	0.339	0.020	0.390	0.009	0.085	0.011
Misspelling Correction	decline i dont fucking think so	0.687	0.120	0.812	0.009	0.174	0.014
Stopword Removal	decline dont fucking think	0.836	0.291	0.850	0.012	0.402	0.028
Stemming	declin dont fuck think	0.800	0.231	0.826	0.012	0.434	0.057
Example 2							
Raw Text	<html> \<*p*>IThinkThe1MillionSalesisTotal BullshktThough</*p*></html>	0.337	0.023	0.427	0.009	0.163	0.018
Lowercasing	<html> \<*p*>ithinkthe1millionsalesistotal bullshktthough</*p*></html>	0.339	0.023	0.419	0.009	0.158	0.019
Special Character Removal	ithinkthe1millionsalesistotalbullshktthough	0.378	0.024	0.537	0.008	0.169	0.010
Word-Boundary Changing	i think the 1 million sales is total bullshkt though	0.573	0.024	0.600	0.009	0.365	0.017
Slang & Leetspeak Removal	i think the 1 million sales is total bullshkt though	0.573	0.024	0.600	0.009	0.365	0.017
Misspelling Correction	i think the 1 million sales is total bullshit though	0.782	0.118	0.845	0.008	0.402	0.018
Stopword Removal	think 1 million sales total bullshit though	0.751	0.075	0.809	0.009	0.372	0.018
Stemming	think 1 million sale total bullshit though	0.786	0.170	0.845	0.009	0.402	0.018

### 4.3 Results analysis

Our results indicate that the proposed novel pre-processing steps, namely word-boundary changes, slang & leetspeak removal, and misspelling correction, significantly improved the detection of toxic text. For instance, in Example 1, the original text contained offensive meaning, but after removing spaces, adding special characters, and introducing typos for interference, the toxicity calculated by Google Perspective API was only 0.21 (and 0.47 by Detoxify API), which could evade screening and be accepted by users. However, our proposed pre-processing step of misspelling correction directly increased all the six indicators, especially the toxicity calculated by the API from 0.210 to 0.687, and the subsequent pre-processing step increased it to 0.80, enabling proper detection and interception.

Furthermore, traditional pre-processing steps sometimes introduced additional interference to toxic text detection. In Example 1, the toxicity calculated by Perspective API decreased after the stemming step, but this did not bring a qualitative change to the toxicity identification of the text. Therefore, we plan to retain the traditional pre-processing steps in our future pre-processing API.

## 5 Conclusion and future work

This paper has presented a comprehensive exploration of the current state of toxic content management on online platforms, highlighting the challenges and potential areas for improvement. The focus has been on the development and evaluation of Specialis Revelio, a novel text pre-processing module designed to enhance the performance of existing toxic text detection tools and services.

Our findings underscore the critical role of data pre-processing in the effective detection and management of toxic content. By implementing novel pre-processing steps, including word-boundary changes, slang and leetspeak removal, and misspelling correction, we have demonstrated a significant improvement in the detection capabilities of the Perspective API. This highlights the potential of our proposed approach to contribute to the creation of safer digital environments.

However, the journey towards a toxicity-free online world is far from over. While our proposed pre-processing module has shown promising results, it also opens up new questions and avenues for future research. For instance, how can we further refine these pre-processing steps to handle more complex forms of toxic content? How can we adapt this approach to different languages and cultural contexts?

Moreover, as we move towards a future where each online platform may have its own self-improving contextual NLP system, the need for effective pre-processing models will only grow. Our proposed solution, which combines a pre-processing module with a toxicity-detection-as-a-service, offers a cost-effective and scalable approach to this challenge. It eliminates the need to develop a toxicity filter from scratch and provides opportunities for continuous improvement and adaptation.

In conclusion, this paper has not only contributed to the ongoing discourse on toxic content management but also provided a practical tool that can be readily implemented and further developed. We hope that our work will inspire further research and innovation in this field, bringing us one step closer to a safer and more inclusive online world.

## CRediT authorship contribution statement

**Johnny Chan:** Conceptualization, Methodology, Writing – review & editing, Supervision. **Yuming Li:** Investigation, Methodology, Validation, Writing – original draft.

## Declaration of Competing Interest

The authors declare that they have no known competing financial interests or personal relationships that could have appeared to influence the work reported in this paper.

## Data Availability

Data will be made available on request.
